# Evolution and revolution of laparoscopic liver resection in Japan

**DOI:** 10.1002/ags3.12000

**Published:** 2017-04-25

**Authors:** Hironori Kaneko, Yuichiro Otsuka, Yoshihisa Kubota, Go Wakabayashi

**Affiliations:** ^1^ Division of General and Gastroenterological Surgery Department of Surgery Toho University Faculty of Medicine Tokyo Japan; ^2^ Department of Surgery Ageo Central General Hospital Saitama Japan

**Keywords:** laparoscopic hepatectomy, laparoscopic liver resection, liver tumor

## Abstract

Due to important technological developments and improved endoscopic techniques, laparoscopic liver resection (LLR) is now considered the approach of choice and is increasingly performed worldwide. Recent systematic reviews and meta‐analyses of observational data reported that LLR was associated with less bleeding, fewer complications, and no oncological disadvantage; however, no prospective randomized trials have been conducted. LLR will continue to evolve as a surgical approach that improves patient's quality of life. LLR will not totally supplant open liver surgery, and major LLR remains to be technically challenging procedure. The success of LLR depends on individual learning curves and adherence to surgical indications. A recent study proposed a scoring system for stepwise application of LLR, which was based on experience at high‐volume Japanese centers. A cluster of deaths after major LLR was sensationally reported by the Japanese media in 2014. In response, the Japanese Society of Hepato‐Biliary‐Pancreatic Surgery conducted emergency data collection on operative mortality. The results demonstrated that mortality was not higher than that for open procedures except for hemi‐hepatectomy with biliary reconstruction. An online prospective registry system for LLR was established in 2015 to be transparent for patients who might potentially undergo treatment with this newly developed, technically demanding surgical procedure.

## 1 Evolution of laparoscopic liver resection

More than 20 years have passed since the first laparoscopic liver resection (LLR). Most early studies in the USA and Europe investigated LLR in benign tumors such as hepatic hemangiomas, adenomas, and cysts.^1^, ^2^ Our center (Division of General and Gastroenterological Surgery, Department of Surgery, Toho University Faculty of Medicine) performed the first laparoscopic partial liver resection for metastatic liver cancer (L‐Mets) and hepatocellular carcinoma (HCC) accompanied by cirrhosis in 1993. The following year, we performed the first laparoscopic left lateral sectionectomy of the liver and found it to be a safe surgical procedure.^3^, ^4^

In the past, indications for LLR were limited tumor size, type, and location. Nodular tumors smaller than 4 cm or pedunculated tumors smaller than 6 cm were considered appropriate candidates. Regarding location, LLR was indicated for tumors in the lower segment and left lateral segment.^5^ After the year 2000, hybrid surgical procedures; namely, laparoscopic‐assisted liver resection and hand‐assisted laparoscopic liver resection commenced, which expanded the indication for LLR.^6^, ^7^, ^8^, ^9^

Further refinement of LLR depended on continued development of surgical equipment for laparoscopy. The difficulty of resecting liver parenchyma—the greatest challenge during LLR—was overcome by the development of suitable laparoscopic instruments and by technical refinements. This new laparoscopic surgical equipment, such as electro‐energy devices, allows complete hemostasis in small hepatic veins, which had previously been difficult, and even in larger vessels. Thus, it facilitated high‐quality hepatic parenchymal dissection in conjunction with effective hemostasis.

At the First Annual Meeting of the Japanese Endoscopic Liver Surgery Study Group in 2007, a detailed questionnaire survey was used to investigate the status and safety of endoscopic liver surgery.^10^ From 2009 to the present time, we have organized hands‐on educational workshops, using a porcine model, which are offered three times a year. These workshops have been attended by more than 600 participants^11^ and helped infiltration of this new technique.

The reports from the first and second Laparoscopic Liver Resection Consensus Conferences, in Louisville (2009)^12^ and Morioka (2014),^13^ show the continuing evolution of LLR. However, although minor liver resection is now standard practice, major liver resection is an innovative procedure still in its exploratory phase.

Despite the benefits of LLR, we must remember that it has considerable technical challenges. Because of the steep learning curve for major LLR,^14^, ^15^ we developed a practical scoring system to assess the difficulty of LLR procedures regularly performed in clinical settings. In addition, a study by Ban and colleagues described a system to determine the difficulty of LLR techniques.^16^

In 2014, sensational media coverage of cluster of deaths after major LLR at a Japanese university hospital increased concerns regarding the safety of LLR. The incident became of great public concern in Japan.^17^ In response, the Japanese Society of Hepato‐Biliary‐Pancreatic Surgery conducted an emergency study of operative mortality. The results showed that, except for hemi‐hepatectomy with biliary reconstruction, mortality was not higher than that for open procedures.^18^

Evidence from large‐scale multicenter Japanese studies using propensity score matching indicated that LLR is superior in the short term and no inferior in the long term to open surgery.^19^, ^20^ From the perspective of professional autonomy, an online prospective registry system for LLR was established in 2015 to help protect patients undergoing this newly developed, technically demanding surgical procedure.^21^

## 2 Surgical procedure

### 2.1 Patient position

The patient is positioned differently based on the location of the resection. Resection of the left hemi‐liver or right anterior region of the liver is performed with the patient in the conventional supine position. The reverse Trendelenburg position improves exposure by gravitationally shifting visceral structures away from the liver. Resection of the right posterosuperior region of the liver should be performed with the patient in the left hemilateral decubitus position, especially for resections requiring mobilization of the right liver from the retroperitoneum.^13^ The prone position may offer better exposure of right posterior segments and lifts the right hepatic vein anterior to the vena cava, which reduces hepatic venous bleeding.^22^ The French position, in which the patient is placed in a supine position with the operating surgeon standing between the spread lower limbs, is advocated by some surgeons.^23^, ^24^

### 2.2 Trocar placement

After pneumoperitoneum is achieved by means of an umbilical incision, the laparoscope is inserted. Trocar placements are a matter of surgeon preference. Briefly, three or four trocars are placed in concentric circles radiating from the tumor to aid operative manipulation during partial hepatectomy. In left lateral sectionectomy, three trocars are placed at the right hypochondrium and bilateral abdomen. For anatomical hepatectomies other than left lateral sectionectomy, four trocars are usually necessary—at the epigastrium, right hypochondrium, and bilateral abdomen.^25^ Intercostal or transthoracic trocars are useful for manipulation during resection of the superior region of the liver.^26^

### 2.3 Pneumoperitoneum

To obtain an operative field for laparoscopic surgery, CO_2_ is generally used to create positive pneumoperitoneal pressure (PP). However, gas embolism is a concern in all types of laparoscopic surgery.^27^, ^28^, ^29^ The overall incidence of gas embolism during laparoscopic surgery is low, approximately 0.15%;^30^ however, when it does develop, the mortality rate is as high as 30%.^31^, ^32^

Because of exposed vessels at the transection plan, the risk of gas embolism during pneumoperitoneum has always been a concern in LLR.^3^, ^33^, ^34^ However, CO_2_ pneumoperitoneum likely reduces bleeding from hepatic veins^35^ and poses few clinical risks, because CO_2_ is more soluble than air in human plasma.^36^ Several clinical studies of LLR suggest that higher PP (18–20 mmHg) can be used to control bleeding during LLR.^37^, ^38^

Bleeding from hepatic veins can be minimized by maintaining low central venous pressure (CVP) during open hepatectomy.^39^, ^40^ Although there is a risk of air embolism caused by absorption of air into the vena cava through the branches of the hepatic veins,^41^ the incidence of clinically significant air embolism is low, and lower CVP is commonly used during open hepatectomy.^42^ It is possible that the risk of gas embolism under pneumoperitoneum is increased by low CVP. Gayet et al.^43^ reported, to reduce the risk of gas embolism, PP should be reduced to the minimum required to maintain a clear operative field (8–10 mmHg) during transection of liver parenchyma. They suggested that the inferior vena cava should be maintained in a ‘half‐filled’ state (i.e. with visible motion in the vein in response to pulse and respiration).

A recent study investigated the relationship of airway pressure, PP, and CVP in experimental LLR.^44^ The authors hypothesized that when airway pressure is high, increasing PP is not effective in controlling the hepatic venous hemorrhage because of increased intrathoracic pressure, bleeding from hepatic veins cannot be controlled under high airway pressure but can be successfully controlled under low airway pressure. On the other hand, under low airway pressure, the risk of pulmonary gas embolism increases when PP is higher than CVP. They concluded that reducing airway pressure is also effective for controlling bleeding from the hepatic vein and safer than increasing PP.

The incidence of gas embolism in major LLR was reported to be 0.2% in a recent review;^45^ however, the pressures used were not causally related to the occurrence of clinically significant gas embolisms. The authors concluded that PP should be maintained at less than 12 mmHg, which appears to be a suitable pressure.

### 2.4 Hepatic parenchymal transection

Prior to liver transection, laparoscopic ultrasound should be performed, to confirm the location of the tumor in relation to the vascular anatomy and to identify other liver lesions.^25^

A wide variety of instruments and maneuvers have been used for liver surgery; however, no single technique has been suggested for laparoscopic, or open, liver parenchymal transection. A review by the Second International Consensus Conference on Laparoscopic Liver Resection (ICCLLR) described the instruments and combinations of instruments to be used, determined by the instrument function and depth of liver resection.^46^ Hepatobiliary surgeons should select techniques based on their level of understanding of the instruments and the applicability of those instruments to particular LLR procedures.

In general, laparoscopic coagulating shears are used to divide the superficial layer of the liver. Deeper transection requires meticulously exposing intraparenchymal structures with an ultrasonic surgical aspirator or clamp‐crushing technique. Vessels with a diameter of 3–7 mm are divided with vessel‐sealing devices or clips. Then, vessels with a diameter of 2 mm or less are diathermically sealed using bipolar sealing devices and then divided. Hemostasis of the resection plane is achieved with monopolar or bipolar cautery. A laparoscopic stapler is used to divide major hepatic vessels, and for simple transection of liver parenchyma with a thickness of 1–1.5 cm.^47^

Parenchymal transection is more hemorrhagic in cirrhotic liver than in non‐cirrhotic liver because of the loss of elasticity due to fibrosis and regeneration of liver tissue, weakness of the altered intrahepatic vasculature, difficulty in identifying intraparenchymal structures and coagulopathy caused by liver dysfunction, portal hypertension, and hypersplenism. Pre‐coagulation technique, in which the resection line is diathermically coagulated using a microwave tissue coagulator or monopolar electrocautery before liver parenchymal transection, can help reduce blood loss during resection of cirrhotic liver or resection without hepatic inflow occlusion.^48^

Reduction of blood loss is essential for successful LLR. Preparation to prevent unexpected hemorrhage, particularly in liver cirrhosis, is the key, as is prompt hemostatic technique. Although controversial in laparoscopic surgery, temporary or intermittent application of Pringle's maneuver—the use of a vessel tape tourniquet or vessel clamp, intra‐ or extracorporeally—can help reduce blood loss during liver parenchymal transection.^13^

From an oncological perspective, optimal segmental territory should be identified before liver transection in anatomical liver resection.^49^ This concept is also applicable in LLR, in which hepatic inflow vessels are isolated with tape traction and occluded using clips or ties. For right or left hemihepatectomy, hilar dissection with individual vessel preparation is standard practice (Video 1).^35^ The Glissonian approach is an important alternative and has been used for all types of anatomical hepatectomies (i.e. hemi‐hepatectomy, sectionectomy, segmentectomy).^50^, ^51^, ^52^, ^53^, ^54^ Surgeons must be mindful to avoid injury to or stenosis of the hepatic duct or Glissonian pedicle, especially when using a stapler to divide the main right or left hilar pedicle by a Glissonian approach.^13^

### 2.5 Hand‐assisted laparoscopic surgery and hybrid technique

Although pure laparoscopy is the most common technique worldwide, there are geographical preferences to use a combination of pure laparoscopic, hand‐assisted laparoscopic surgery (HALS), and hybrid technique in selected cases.^6^, ^7^, ^8^, ^55^ Although current evidence does not indicate which of these approaches is best, HALS and hybrid method are claimed by their proponents to be beneficial for large lesions,^9^, ^56^ posterior lesions^57^, ^58^ and donor hepatectomy.^59^, ^60^, ^61^, ^62^, ^63^, ^64^ HALS and the hybrid method can be used to manage intraoperative difficulties and can theoretically decrease the frequency of conversion to full open incision. These approaches can also be used to train surgeons in major LLR techniques.^6^, ^14^, ^65^

### 2.6 Surgical navigation

It is not an exaggeration to say that the modern era of liver surgery began with intraoperative use of ultrasonography.^66^ Preoperative simulation using three‐dimensional (3D) reconstructions of intrahepatic structures, segmentation, and volumetric measurements may improve surgical planning and intraoperative navigation of hepatectomy, including laparoscopic procedure; however, evidence for this is limited.^67^ A recent modality, laparoscopic near‐infrared fluorescence imaging, appears to be useful for intraoperative detection of tumor extension and precise anatomical resection.^68^ (Video 2).

## 3 Technical considerations

### 3.1 Learning curve

The learning curve for LLR is steep; however, it is believed that LLR is reproducible.^14^ Experienced liver surgery units must help standardize the techniques required for each LLR procedure^25^ and share their experience of the technical challenges of LLR. Major LLR requires a high level of technical skill and has a steeper learning curve than minor LLR.^15^ One study found that 60 laparoscopic minor liver resections was adequate experience before attempting major LLR.^69^ Specific training in advanced laparoscopy is also required.^14^

### 3.2 Scoring of LLR difficulty

Because of the wide variety of LLR procedures and the steep learning curve, the technical ability of surgical teams should be assessed. A recent study described a difficulty scoring system for stepwise application of LLR, which was based on experience at high‐volume Japanese centers.^16^ The proposed system estimates surgical difficulty by tumor location, extent of liver resection, tumor size, proximity to major vessels, and existing chronic liver damage. Difficulty is classified as low, intermediate, advanced, or expert, and the system can be used to select patients according to the skill of the surgeon.

### 3.3 Training

The Second ICCLLR described the urgent need to identify the skills required by trainees and practicing surgeons.^13^ There is no established training strategy for LLR; however, the Japanese Endoscopic Liver Surgery Study Group, with support by the Japan Society for Endoscopic Surgery (JSES), developed a unique training program.^11^ This 2‐day course comprises academic and technical lectures, a video clip, and an experimental laboratory that includes study of animal liver anatomy and small‐to‐large laparoscopic partial hepatectomies, left lateral sectionectomy, hand‐assisted procedure, and hepatic hilar dissection. The key points of the course are techniques to avoid, appropriate use of instrumentation, maintenance of the operative field, and meticulous isolation of intrahepatic vasculatures. As of 2015, more than 600 participants, mainly young Japanese surgeons, have taken the course.

### 3.4 Skill qualification

Experience in both laparoscopic surgery and open liver resection is necessary for successful LLR. The Louisville Statement agreed that LLR should be performed only at centers with combined expertise in liver and laparoscopic surgery,^12^ but did not provide detailed criteria for defining such expertise.

An endoscopic surgical skill qualification system was developed by JSES and has been used since 2004. Various minimally invasive surgical procedures can be evaluated by submitting unedited full‐length videos of procedures. LLR was specified as a laparoscopic procedure that should be evaluated using this system.^70^ Partial LLR with isolation and division of intrahepatic vessels has been the procedure required for qualification. Evaluation includes assessment of items common to digestive surgery (60 points) and items specific to liver surgery (40 points). A score higher than 70 is considered passing. Assessment of common items includes fundamental techniques, such as progress of the surgery, exposure of the operative field, and selection and use of instruments. Liver‐specific assessment mainly evaluates use of ultrasound, techniques for parenchymal transection (from hemostasis to intrahepatic vessel transection), and specimen retrieval. The effort of this system on patient safety and further expansion of LLR needs to be carried out.

## 4 Indications and oncological outcomes

### 4.1 Laparoscopic liver resection for benign liver tumors

In 1991, Reich et al. performed LLR for the first time as a method for partial resection of benign tumors.^1^ After its dissemination, LLR has since been performed proactively for benign tumors with favorable outcomes.^2^, ^71^ It is often difficult to differentiate HCC from hepatocellular adenoma or focal nodular hyperplasia^72^, ^73^ and needle biopsy has this suggests that there is a risk of implantation in cases of malignant tumor.^74^ In addition, previous studies reported substantial symptom improvement after liver resection for symptomatic patients^75^ and that LLR was useful for diagnosis and treatment.^76^ Occasionally, surgical treatment is indicated for hepatocellular adenoma and hepatic hemangioma because these lesions sometimes progress to form bulky tumors and present a risk of rupture. Herman et al.^77^ safely carried out LLR, with low rates of morbidity and mortality, in 31 patients with hepatocellular adenoma, even though approximately half of the patients had tumors measuring 8 cm or larger. They concluded that hepatocellular adenoma is an extremely good indication for LLR. Bai et al.^78^ reported using a modified LLR method for 8–12‐cm hepatic cavernous hemangiomas. An electromechanical morcellator was used in an ingenious attempt to minimize the surgical wound, alleviate postoperative pain, and reduce the duration of postoperative hospital stay. Liver resection is less suitable for benign liver tumors than for malignant tumors, but LLR can greatly assist in diagnosis and treatment when it is difficult to differentiate benign from malignant tumors, when patients have symptoms, and when there is a risk of rupture or bleeding.

### 4.2 Laparoscopic liver resection for malignant liver tumors

#### 4.2.1 Hepatocellular carcinoma

In regards to malignant liver tumors, LLR is most often indicated for HCC, followed by L‐Mets. In 2005, Kaneko et al.^79^ described the utility of LLR for HCC. A later systematic review and meta‐analysis of LLR and open liver resection (OLR) showed no significant difference in surgical curability, but LLR was associated with less invasive than OLR.^80^, ^81^, ^82^ These reports are considered the main evidence supporting the utility of LLR in the setting of HCC, because it is unrealistic to conduct a randomized controlled trial comparing the outcomes of OLR and LLR. In a recent multicenter study, Takahara et al.^19^ used propensity score matching to compare treatment outcomes between 446 patients who underwent LLR and 2969 patients who underwent OLR. LLR resulted in significantly less bleeding, shorter hospital stay, and fewer complications, with no difference in survival rates. In general, HCC, which is often accompanied by cirrhosis, has a high risk of postoperative complications such as ascites and liver failure.^83^ A meta‐analysis comparing the outcomes of LLR and OLR for HCC in patients with chronic liver disease reported favorable short‐term outcomes in the LLR group,^84^ which suggests that LLR results in fewer postoperative complications owing to factors such as less bleeding, a simpler mobilization procedure, and minimal destruction of the body wall.^48^ Furthermore, anatomical resection is preferred when performing curative resection for HCC in patients with good functional liver reserve.^85^ As the number of LLR cases increases, so, too, does the number of reports on major LLR.^86^, ^87^ The evidence indicates that LLR is safe when performed by surgeons with sufficient experience. In addition, laparoscopic segmentectomy may be suitable for cases involving technically challenging posterosuperior segments and segment I, if surgeons improve their skills and exercise ingenuity.^26^, ^88^, ^89^

From an oncological perspective, it is possible to use an LLR approach that is non‐inferior to conventional anatomical resection. In a study of major LLR and the conventional open approach (OLR), Komatsu et al.^90^ investigated 38 matched patients with HCC in each group and found that the 3‐year survival rate of 73.4% in the LLR group did not significantly differ from the rate in the OLR group. Lee et al.^91^ performed LLR for HCC in the posterosuperior or anterolateral segments. The 5‐year survival rate was 88.5% in the posterosuperior group, and there was no significant difference in short‐ or long‐term surgical outcomes between the posterosuperior and anterolateral groups when LLR was performed by experienced surgeons. These results show that technological advances in laparoscopic anatomical resection for HCC have made it oncologically feasible to perform LLR. Indeed, accumulating data from relatively high‐quality studies with long follow‐up periods show the non‐inferiority of LLR as compared with OLR in relation to invasiveness, safety, and long‐term outcomes.

#### 4.2.2 Liver metastases

In liver metastases (L‐Mets), surgical indications are consistent with the oncological features of the original tumor,^92^ but this applies mostly to colorectal cancer. In general, partial resection is the standard surgical procedure for metastatic liver cancer because curative treatment is achieved by performing minimized liver resection with margin.^93^ This understanding led to studies of the effectiveness of LLR, for which partial resection is the best surgical indication.^3^ In a multicenter study of 1331 patients with liver metastasis of colorectal cancer, Beppu et al.^20^ used propensity score matching to compare LLR and OLR and found no significant intergroup differences in rates of complications, mortality, survival, or recurrence‐free survival and reductions in estimated blood loss and duration of hospital stay in the LLR group. Allard et al.^94^ separately matched operative risk factors and prognostic factors and compared short‐ and long‐term treatment outcomes between LLR and OLR. The LLR group had fewer severe complications and shorter postoperative hospital stays, with no significant intergroup differences in 3‐ or 5‐year survival rates. In contrast, Tranchart et al.^95^ compared 89 matched patients who underwent concurrent laparoscopic resection of L‐Mets and the primary cancer with 89 patients who underwent concurrent open resection. Among patients in fair general condition with relatively few, small tumors, concurrent laparoscopic resection was safe and achieved long‐term outcomes comparable to those achieved by open surgery. In recent years, treatment with curative intent is actively performed by making modifications to liver resection and perioperative chemotherapy for unresectable liver metastases,^96^, ^97^ which has increased awareness of the utility of LLR as part of multidisciplinary therapy. Chemotherapy administered before liver resection may cause liver damage^98^, ^99^ and is considered disadvantageous for LLR. However, LLR can still be performed safely if the surgeon understands the pharmacological profile of the chemotherapeutic agents, chooses appropriate surgical instruments, and perform the proper surgical maneuvers.^100^ A study of the utility of two‐stage hepatectomy for bilobar multiple liver metastases as proactive curative surgical treatment for unresectable liver metastases^101^ found that two‐stage LLR drastically reduced development of intra‐abdominal adhesions after first‐stage liver resection and thus simplified second‐stage liver resection, which is considered technically difficult, thereby achieving long‐term outcomes comparable to those for open two‐stage hepatectomy.^102^, ^103^

#### 4.2.3 Recurrent tumor in HCC and L‐Mets

Re‐hepatectomy is thought to be useful for improving long‐term survival in recurrent HCC and L‐Mets;^104^, ^105^, ^106^ however, such procedures can be technically challenging because of conditions created by the first surgery.^107^ As mentioned earlier, when performed as the first surgery, LLR is associated with minor, or no, adhesion in the abdominal cavity, except at resection margins in the liver. This makes it a useful treatment strategy, especially for patients likely to undergo re‐hepatectomy.^26^

#### 4.2.4 Hilar bile duct cancer

Few studies have investigated LLR and bile duct reconstruction surgery in patients with cancers of the upper bile duct, such as hilar bile duct cancer;^108^, ^109^, ^110^ thus, it is difficult to evaluate the utility and safety of these procedures. Lymph node dissection and organ reconstruction are unnecessary in LLR for HCC and metastatic liver cancer, and this appears to be important for the safety and surgical curability of LLR. Indications for LLR should be carefully re‐evaluated, because mortality is significantly higher for lobectomy accompanied by bile duct resection (9.76%) compared to lobectomy alone (1.56%).^18^

## 5 The two consensus conferences

The first ICCLLR was held in Louisville in 2008.^12^ Forty‐five experts in liver surgery were invited to discuss the status of laparoscopic liver surgery, and this was the first opportunity for liver surgeons performing LLR to meet in person. The Louisville consensus statement recommends that solitary tumors measuring 5 cm or less located in the peripheral liver at segments 2–6 are good candidates for LLR.^12^ Since then, the number of LLRs performed worldwide has increased exponentially,^111^ and LLR has expanded to include minor resection at difficult sites,^52^ major resection,^86^, ^112^, ^113^ robotic hepatectomy,^114^ parenchymal‐sparing anatomical resection, and donor hepatectomy.^59^ More than 9500 LLR procedures have been reported worldwide.^115^ Six years after the Louisville Consensus Conference, the second ICCLLR was held on 4–6 October 2014, in Morioka, Japan, to better define the role of LLR and develop internationally accepted guidelines.^13^

During the 6 years between these consensus conferences, this relatively new surgical technique has evolved and is rapidly being adopted worldwide. The main goal of the chairman of the second conference was to facilitate collaboration among liver surgeons worldwide.^116^ The conference concluded that:

1. LLR is superior to OLR because the laparoscope allows better exposure with a magnified view, and PP reduces hepatic vein bleeding from the cut surface.^35^
2. The concept of liver resection has changed from an open ventral approach to a laparoscopic caudal approach. The laparoscopic caudal approach allows important structures, such as the hilar plate and vena cava, to be clearly imaged and presented in front of the surgeon.^35^

The Morioka Consensus Conference used an independent jury‐based consensus model to achieve its goal through analysis of the available literature and expert presentations to jury panels.^117^ Because the evidence level for LLR is low for developing strong recommendations, it was reasonable to develop consensus statements by jury decision. Forty‐three liver surgeons, including 34 expert panelists and nine jury members not directly involved in LLR, were invited from 18 countries. The Morioka Consensus Conference attempted to answer three central questions: (i) What are the comparative short‐term and long‐term outcomes of LLR versus OLR? (ii) What are the indications with respect to the difficulty of LLR? and (iii) What is needed in order to improve the quality of LLR?

The organizing committee prepared 17 questions related to the benefits and techniques of LLR, and 17 working groups were assigned to answer these questions by means of extensive literature reviews. The jury provided recommendations for the first seven questions, which were related to the benefits and risks of LLR. The experts provided recommendations for the next 10 questions, which were related to technical aspects of LLR. Expert recommendations were created from the expert presentations, assessment of the literature, and experience in particular techniques. Table 1 summarizes the jury and expert recommendations.

**Table 1:** Summary of recommendations of Morioka Consensus Conference

**Jury Recommendations** MINOR LLR is confirmed to be a standard practice in surgery but is still in the assessment phase (IDEAL 3) as it is adopted by more surgeons. Some outcomes, such as certain postoperative complications and duration of stay, were superior to those of open procedures; no outcomes were inferior. The quality of studies is generally LOW. Additional higher‐quality studies are needed in order to define the role and benefits of minor LLR in relation to open surgery. MAJOR LLR is an innovative procedure. It is still in the exploratory, learning phase (IDEAL 2b) and has incompletely defined risks. It should continue to be introduced cautiously. Duration of stay was shorter than that of open procedures; other outcomes were non‐inferior. The quality of studies is generally LOW. There is an urgent need for additional higher‐quality studies and registries, to define the role and benefits of major LLR in relation to open surgery. LAPAROSCOPIC DONOR SURGERY Pediatric donor surgery is classified as stage IDEAL 2b, as is major laparoscopic liver surgery. Adult‐to‐adult donor surgery is an innovative procedure still in the development phase (IDEAL 2a). The recommendation is that laparoscopic donor surgery be carried out under institutional ethical approval and with registry reporting. EDUCATION MAJOR laparoscopic liver surgery requires considerable technical skill and has a steep learning curve. Skill acquisition by trainees and practicing surgeons should be the subject of an urgent, focused effort by leaders in this field. The future of laparoscopic liver surgery depends on education initiatives. DIFFICULTY SCORING SYSTEM A scoring system is being developed to grade the technical difficulty of laparoscopic liver surgery and safely guide development of expertise. Validation and application of this process is STRONGLY recommended.
**Expert Technical Recommendations** There is GENERAL AGREEMENT that experience in both open liver surgery and advanced laparoscopy is mandatory and that surgeons must begin with minor laparoscopic resections. HALS AND HYBRID TECHNIQUE can help overcome certain difficulties associated with pure LLR and may be useful in minimizing conversions. CONCEPTUAL CHANGES include: A caudal approach that optimizes hilar dissection and transection of the liver parenchyma for major and/or anterior resections. A lateral approach (left lateral decubitus position) that optimizes access to posterior segments. CO_2_ PNEUMOPERITONEUM of 10–14 mmHg is generally used along with low central venous pressure. This provides satisfactory control of back bleeding during liver transection. Selective control of inflow during laparoscopy may be more efficient than during open surgery (a possible effect of pneumoperitoneum). Careful inspection should be routinely carried out after decreasing pneumoperitoneum pressure. LAPAROSCOPIC PARENCHYMAL TRANSECTION requires specific instruments. This provides for satisfactory control of back bleeding during liver transection. Surgeons must have a concrete understanding of the advantages and limitations of available instruments to ensure safe and effective LLR. Deeper transection should be carried out meticulously by exposing intraparenchymal structures with an ultrasonic aspirator (Cavitron ultrasonic surgical aspirator or equivalent), clamp‐crushing technique, or similar parenchymal dissection technique. ENERGY DEVICES are efficient and reliable. Despite their benefits, energy devices cannot replace acquisition of basic skills of hepatic surgery such as meticulous dissection, direct visualization, and sealing of vascular structures. The argon beam coagulator is not generally recommended because of the risk of gas embolism. The HILAR APPROACH includes individual hilar dissection and the Glissonian approach. Hilar dissection cannot be carried out distal to the first bifurcation of the portal branch (i.e. the right anterior and posterior sectional branches). The Glissonian approach is an important alternative when used appropriately. ANATOMICAL RESECTION for HCC and margin‐negative parenchyma‐sparing resection for colorectal cancer liver metastases are standard‐of‐care procedures. The laparoscopic versions of these techniques need to be standardized to increase uptake. Use of intraoperative ultrasound is recommended for determining the accuracy of clear margins and avoiding injury to major pedicles during LLR.

HALS, hand‐assisted laparoscopic surgery; LLR, laparoscopic liver resection.

A major achievement of these two consensus conferences was that all international experts were present in the same room at the same time. These expert technical recommendations will never be confirmed by level 1 evidence, but still need to be shared so that beginners can benefit from expert guidance. Another major achievement of the consensus conferences is the publication activities related to the conferences and the systematic reviews prepared to develop recommendations before the Morioka Consensus Conference.^46^, ^67^, ^84^, ^118^, ^119^, ^120^, ^121^, ^122^ We hope that all these publications will contribute to the safe uptake of LLR. Finally, the most important message from the Morioka Consensus Conference was the need to protect patients undergoing this new surgical procedure. We recommended a broad‐based registry because, even though minor LLR is now standard practice, major LLR remains an exploratory procedure. In accordance with the recommendations, we launched an online prospective registry system for LLR in October 2015 in Japan^21^ and are now preparing a worldwide registry. Furthermore, we developed a scoring system to define the difficulty of LLR, similar to the Child–Pugh score, so that beginners can start performing LLR easily and safely.^16^ Selection of appropriate patients in accordance with the surgeon's skills will protect patients. Difficult cases should be deferred, depending on the surgeon's LLR learning curve.

## 6 Online registry system

The cluster of deaths after major LLR procedures at a Japanese university hospital was sensationally reported just after the Morioka Consensus Conference and highlighted the need for safe introduction of major LLR.^21^ Several deaths after major laparoscopic liver resections at a regional cancer center in Japan were reported during the same period. After media raised concerns about LLR safety, the Japanese Society of Hepato‐Biliary‐Pancreatic Surgery (JSHBPS) conducted an urgent data collection on operative mortality for LLR among more than 200 board‐certified training centers in Japan, the results of which were released at a press conference on 23 March 2015. Data were presented on deaths within 30 and 90 days after LLR during 2011 to 2014. The results clearly showed that operative mortality after LLR had not increased, even though the number of cases per year had gradually risen, and that mortality was not higher than that for open procedures, except for hemi‐hepatectomy with bile duct resection.^21^ In response to the sensational media coverage, we launched the online prospective registry system for LLR as an act of professional autonomy.^21^ All member institutions of the Japanese Study Group of Endoscopic Liver Surgery (JSGELS) and all board‐certified JSHBPS training centers are expected to participate in this online registry. LLR operators are requested to enter relatively simple items online at four time points: preoperatively, postoperatively, after discharge, and after readmission. We have obtained the latest data prospective registry over the past year. In 1784 total cases, operative mortality for 30 days and 90 days was 0.11% and 0.22%, respectively. Major LLR, which consists of operative hemi‐hepatectomy, sectionectomy and subsegmentectomy, operative mortality for 30 days and 90 days was 0.53% and 1.06%, respectively (Table 2).^123^, ^124^ We expect that this will become one of the largest prospective databases of LLR in the world and that it will serve to protect patients by accurately assessing the outcomes.

**Table 2:** Operative mortality of latest data prospective registry in Japan^a^

Mortality	
Cases of partial resection, left lateral sectionectomy, segmentectomy, sectionectomy and hemihepatectomy	
30‐day mortality rate	90‐day mortality rate
0.11%	0.22%
(2/1784)	(4/1784)
Mortality	
Cases of segmentectomy (except left lateral sectionectomy), sectionectomy and hemihepatectomy	
30‐day mortality rate	90‐day mortality rate
0.53%	1.06%
(2/376)	(4/376)

a October 2015 to December 2016.

## 7 Insurance reimbursement for LLR in Japan

One of the main purposes for creating JSGELS was to achieve insurance reimbursements for LLR under the national health insurance system. Under the financial year (FY)2010 revisions to reimbursements for treatment in Japan, LLR is no longer categorized as an advanced medical treatment and is therefore covered for the first time. Revisions to reimbursements take place every 2 years in Japan. The strict facility criteria were lifted in FY2012, and the FY2014 revisions increased the reimbursement for partial hepatectomy to 59 680 points (10 points = approximately US$1); 74 880 points were allotted to left lateral sectionectomy, as before. These reimbursements were around 23 000–28 000 points ($2100 to $2600) higher than reimbursements for open partial resection (36 340 points) and left lateral sectionectomy (46 130 points). The national health insurance covers ‘procedural costs’ for LLR, including the cost of surgical instruments used only for this procedure, but, given the shorter hospital stay needed for patients treated by LLR, the level of reimbursement is likely to spur even more widespread use.

We conducted two multicenter studies that used propensity score matching to compare perioperative and long‐term outcomes of LLR and OLR for HCC^19^ and colorectal liver metastases (CRLM).^20^ Data were collected from more than 30 Japanese centers where patients underwent resection for HCC or CRLM; more than 4700 patients were analyzed in these studies. To date, these two reports are the largest published studies to use propensity score analysis of LLR for HCC or CRLM.^125^ In addition, they were used to show the efficacy and safety of LLR for the FY2016 revision to reimbursements for LLR.

From 1 April 2016, all LLR, except LLR with bile duct resection, were eligible for reimbursement. As shown in Table 3, the reimbursements for LLR are around 23 000–77 000 points ($2100 to $7000) higher than reimbursements for OLR. These differences are attributable to data we submitted, which show less blood loss, decreased complication rates, and shorter hospital stay for LLR as compared with OLR. Strict facility criteria have been established, and use of the online prospective registry system for LLR is mandatory for reimbursement. We estimate that approximately 200 centers in Japan receive reimbursements for all LLR procedures. JSGELS and JSHBPS encourage the safe introduction of major LLR to these institutions.

**Table 3:** FY2016 revision to reimbursements for open liver resection (OLR) and laparoscopic liver resection (LLR) in Japan

Extent of liver resection	OLR	LLR
Partial resection	36 340 pts ($3300)	59 680 pts ($5400)
Left lateral sectionectomy	46 130 pts ($4200)	74 880 pts ($6800)
Subsectionectomy	56 280 pts ($5100)	108 820 pts ($9900)
One sectionectomy	60 700 pts ($5500)	130 730 pts ($11 900)
Two sectionectomy	76 210 pts ($6900)	152 440 pts ($13 900)
Three sectionectomy	97 050 pts ($8800)	174,090 pts ($15 800)

FY2016, financial year 2016; $, USD.

## 8 Conclusion

Recognition of LLR as a standard surgical method is increasing. Uptake of LLR will be facilitated by mastery of surgical skills, compliance with LLR indications, and maintenance of minimal invasiveness and safety. These are the fundamental principles of laparoscopic surgery.

## Conflicts of Interest

Authors declare no conflicts of interest for this article.

## Supporting information


**Video 1.** Laparoscopic right hemihepatectomy, http://www.wiley.co.jp/journals/AGS1-1_Kaneko_Video1.html

**Video 2.** Laparoscopic S2 segmentectomy, http://www.wiley.co.jp/journals/AGS1-1_Kaneko_Video2.html
